# Everolimus (EVE) and exemestane (EXE) in patients with advanced breast cancer aged ≥ 65 years: new lessons for clinical practice from the EVA study

**DOI:** 10.18632/oncotarget.25874

**Published:** 2018-08-07

**Authors:** Marina Cazzaniga, Claudio Verusio, Mariangela Ciccarese, Alberto Fumagalli, Donata Sartori, Cristina Ancona, Mario Airoldi, Gabriella Moretti, Corrado Ficorella, Valentina Arcangeli, Lucrezia Diodati, Alberto Zambelli, Antonio Febbraro, Daniele Generali, Mirco Pistelli, Ornella Garrone, Antonino Musolino, Patrizia Vici, Michela Maur, Lucia Mentuccia, Nicla La Verde, Giulia Bianchi, Salvatore Artale, Livio Blasi, Matilde Piezzo, Francesco Atzori, Anna Turletti, Chiara Benedetto, Maria Concetta Cursano, Alessandra Fabi, Vittorio Gebbia, Antonio Schirone, Raffaella Palumbo, Antonella Ferzi, Antonio Frassoldati, Claudio Scavelli, Luca Clivio, Valter Torri on behalf of The EVA Study Group

**Affiliations:** ^1^ Research Unit Phase I trials, ASST Monza, Monza, Italy; ^2^ Oncology Unit, ASST Monza, Monza, Italy; ^3^ Oncology Unit, ASST della Valle Olona-Presidio Ospedaliero di Saronno, Saronno, Italy; ^4^ Oncology Unit, Ospedale "Vito Fazzi" di Lecce, Lecce, Italy; ^5^ Oncology Unit, Ospedale Moriggia Pelascini, Gravedona, Italy; ^6^ Oncology Unit, AULSS 3, Mirano, Italy; ^7^ Oncology Department, Policlinico di Palermo Paolo Giaccone, Palermo, Italy; ^8^ Oncology Unit 2-Città della Salute e della Scienza di Torino, Torino, Italy; ^9^ Oncology Unit, IRCCS Arcispedale S. Maria Nuova, Reggio Emilia, Italy; ^10^ Dipartimento di Scienze Cliniche Applicate e Biotecnologiche (DISCAB)-Università Degli Studi Dell’Aquila, L’Aquila, Italy; ^11^ Oncology Unit Rimini Azienda USL Romagna, Rimini, Italy; ^12^ Oncology Unit 2, Azienda Osp edaliera Universitaria Pisa via Roma 67, Pisa, Italy; ^13^ Oncology Unit, ASST Papa Giovanni XXIII, Bergamo, Italy; ^14^ Oncology Unit, Ospedale Sacro Cuore di Gesù, Fatebenefratelli, Benevento, Italy; ^15^ Brest Unit, ASST di Cremona, Cremona, Italy; ^16^ Oncology Unit, AOU Ospedali Riuniti Umberto I-GM Lancisi-G Salesi, Ancona, Italy; ^17^ Oncology Unit, AOS Croce e Carle Ospedale di Insegnamento, Cuneo, Italy; ^18^ Oncology Unit, Azienda Ospedaliero-Universitaria di Parma, Parma, Italy; ^19^ Oncology Unit 2, Istituto Nazionale Tumori Regina Elena–IFO, Roma, Italy; ^20^ Oncology Unit, Policlinico University Hospital of Modena, Modena, Italy; ^21^ Oncology Unit, ASL di Frosinone Osp. "SS. Trinità", Sora, Italy; ^22^ Oncology Unit, ASST Fatebenefratelli Sacco Presidio Ospedaliero Fatebenefratelli, Milano, Italy; ^23^ Oncology Unit 1, Fondazione IRCCS Istituto Nazionale Tumori, Milano, Italy; ^24^ Oncology Departement, Ospedale di Gallarate ASST Valle Olona, Gallarate, Italy; ^25^ Oncology Unit, ARNAS Civico Palermo, Palermo, Italy; ^26^ National Cancer Institute "Fondazione Giovanni Pascale", Napoli, Italy; ^27^ Struttura Complessa di Oncologia Medica Azienda Ospedaliero-Universitaria Cagliari, Italy; ^28^ Oncology Unit, Ospedale Martini della ASL Città di Torino, Torino, Italy; ^29^ Dipartimento Universitario Ginecologia e Ostetricia 1, Ospedale S. Anna Torino, Turin, Italy; ^30^ Oncology Unit, Università Campus Bio-Medico, Roma, Italy; ^31^ Oncology Unit 1, Istituto Regina Elena–IFO, Roma, Italy; ^32^ Oncology Unit, Ospedale La Maddalena, Palermo, Italy; ^33^ Oncolgy Department, Istituto Scientifico Romagnolo per lo Studio e la Cura dei Tumori (IRST) IRCCS, Meldola, Italy; ^34^ Oncology Unit, IRCCS ICS Maugeri, Pavia, Italy; ^35^ Oncology Unit, ASST OVEST Milanese, Presidio di Legnano, Legnano, Italy; ^36^ Oncology Unit, Az Ospedaliero Universitaria di Ferrara, Ferrara, Italy; ^37^ Oncology Unit, Ospedale "S. Cuore di Gesù", Gallipoli, Italy; ^38^ IRCCS-Istituto di Ricerche Farmacologiche “Mario Negri”, Milano, Italy

**Keywords:** elderly, everolimus, exemestane, hormone-receptor positive, advanced breast cancer

## Abstract

**BACKGROUND:**

The present analysis focuses on real-world data of Everolimus-Exemestane in advanced HR+ve, HER2-ve elderly breast cancer patients (aged 65 years) included in the EVA study, with unique findings in those aged 70 years.

**METHODS:**

Data are collected from clinical records and analysed according to age cut-off (< 65 years; 65 - 69 years and {greater than or equal to} 70 years). Relationship of analyzed variables with response were tested by mean of a Mantel-Haenszel chi square test. Time to event analysis was described by Kaplan Meier approach and association with baseline characteristics was analysed by stratified log-rank test and proportional hazard model.

**RESULTS:**

From July 2013 to December 2015, the EVA study enrolled overall 404 pts. 154 patients out of 404 (38,1%) were aged {greater than or equal to} 65 years, of whom 87 were {greater than or equal to} 70 years. Median duration of EVE treatment was 28.5 weeks (95% CI 19.0 - 33.8) in patients aged 65-69 years and 24,4 weeks (95% CI 19,2 - 33,2) in those aged {greater than or equal to} 70 years. Fewer patients aged 65 years received the highest EVE Dose-Intensity (>7.5 mg/day) in comparison to younger patients (49,6% vs. 66,8%). Grade 3–4 toxicities occurred to 55 patients (35,7%), mainly stomatitis (10,9%), rash (5,8%) and non-infectious pneumonitis (NIP) (3,6%). Some toxicities, such as weight loss and anaemia were peculiarly observed in patients aged {greater than or equal to} 70 years. Five treatment-related deaths were collected (3,2%).

**CONCLUSIONS:**

EVE-EXE combination remains one of the potential treatments in HR+ patients also for elderly ones.

## INTRODUCTION

Elderly patients with cancer are usually under-represented in clinical trials [1] [2], especially if older than 70 years of age, resulting in a lack of evidence on how they should be treated.

Drug elimination pathways, effects of ageing on renal function, and empiric dose adjustments made are to be taken into account [1] [3].

Even if endocrine therapy (ET) is still considered the treatment of choice for patients with hormone-receptor-positive (HR+), human epidermal growth factor receptor 2-negative (HER2−) advanced breast cancer (ABC) in metastatic setting independently of age [3], there is growing evidence that the association of ET with a target agent, such as the mTOR inhibitor Everolimus (EVE) or the cyclin-dependent kinase 4/6 inhibitors (CDK 4/6-Is) significantly prolongs Progression-Free-Survival (PFS), increases Overall Response Rate (ORR), generally determining an improvement in clinical outcome [4] [5] [6].

However, most of these trials do not report specific details regarding the outcome and the main toxicities in elderly patients, who in any case represent two-thirds of the population affected by breast cancer.

The 5-year relative survival rate is 20% for all patients with ABC and worse (≤20%) for those older than 65 years [7].

The BOLERO-2 trial demonstrated that adding EVE to Exemestane (EXE) improved progression-free survival (PFS) while maintaining quality of life when compared with EXE alone [4]. Because many women with HR+ve advanced breast cancer are elderly, the tolerability profile of EVE plus EXE in this population is of particular clinical interest and was explored in a pre-specified analysis of the main study in patients aged ≥ 65 years and as an exploratory analysis in those aged ≥ 70 years [8].

The EVA study is a multicentre, longitudinal, retrospective study which reported the outcome of ABC patients treated with the combination of EVE and Exemestane (EXE) in a real-life setting [9]. The improved safety and efficacy profile for EVE-EXE combination compared with EXE alone suggests that this strategy could be a valid option of treatment after a first-line therapy with Overall Response Rate (ORR)[4] [9], as well as in patients previously treated with high-dose(HD) Fulvestrant, as demonstrated by our previously reported results.

The present analysis focuses on real-world data regarding the EVE-EXE combination in a group of elderly ABC patients (aged ≥ 65 years) included in the EVA study, by highlighting simultaneously some peculiar findings in those aged ≥ 70 years, who are of special interest in the clinical practice, and with the attempt of performing some comparisons between old and young ABC patients.

## RESULTS

### Patient and tumour characteristics

**Figure 1:**
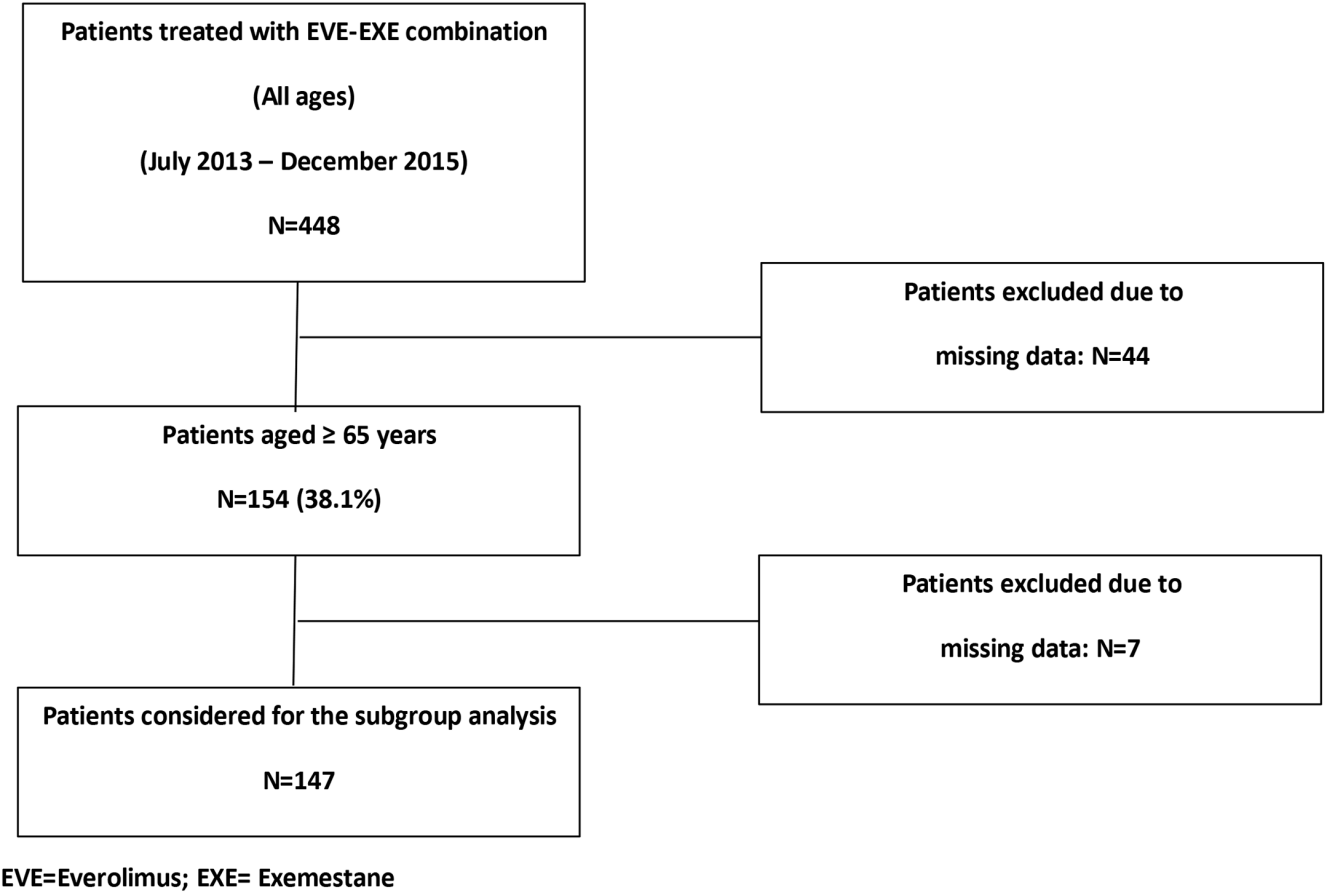
CONSORT flow chart

We overall retrospectively retrieved clinical data from 448 patients with ABC treated with the EVE-EXE combination between July 2013 and December 2015, of whom 154 (38,1%) were aged 65 years or more, including 87 very old patients (70 years or more). Data from 44 records did not satisfy the pre-specified criteria of the main study and were excluded from the primary analysis, whereas 7 patients out of 154 (4,5%) aged ≥ 65 years were excluded from the present analysis because of lack of useful data (Figure 1).

Median follow up time was 29,2 months (26,8 – 30,8). Median age at the time of study enrolment was 70 years (65 – 83). ECOG Performance Status was 0-1 in the majority of patients, without any significant difference among the three groups (p=0.57).

At the time of first relapse, a statistically significant positive trend was detected for some types of comorbidities and age, especially for cardiac/vascular (p=0.0001) and musculo-skeletal (p=0.0082) ones.

All patients had HR+ tumours (ER+ = 145, 94.2%; PgR+ve = 132, 85.7%). The majority of the patients had one (55.2% and 70.1%, according to age cut-off) metastatic site, mainly at bone level (68.7% and 60.9%, according to age cut-off). No statistically significant difference was found between younger and older patients in terms of number of metastatic sites (p=0.72). All the patients enrolled had received prior therapies for their metastatic disease before EVE treatment, mainly chemotherapy followed by endocrine treatment (53.7%) in those aged 65-69 years and endocrine therapy alone (47.1%) in those aged ≥ 70 years. Interaction between age and type of previous therapy was not significant (p=0.02). Median number of previous treatments was 2 (1 – 6). Key prior anti-neoplastic treatments before EVE starting were: Fulvestrant (81, 52,6%), AIs as last therapy (60, 38,9%) and chemotherapy (13, 8,4%).

Details are summarized in Table 1.

**Table 1:** Patients’ and disease characteristics

	Patients, %			
	Age < 65 N=249 (%)	Age 65 - 69 N=67 (%)	Age ≥ 70 N=87 (%)	P value^*^
**ECOG PS**				
0 - 1	247 (98.8)	67 (100.0)	85 (97.7)	0.57
2	3 (1.2)	-	2 (2.3)	
**Hormone Receptor Status**				
ER+	231 (92.8)	63 (94.0)	82 (94.3)	p=0.60
PgR+	211 (84.7)	58 (86.6)	74 (85.1)	p=0.88
**Comorbidities**				
Cardio-vascular	70 (28.1)	37 (55.2)	41 (47.1)	0.0001
Metabolic	40 (16.0)	16 (23.8)	18 (20.7)	0.2180
Gastrointestinal	20 (8.0)	3 (4.5)	10 (11.5)	0.4630
Musculoskeletal	11 (4.4)	5 (7.5)	11 (12.6)	0.0082
Others	43 (17.3)	20 (29.8)	29 (33.3)	0.0008
**Number of metastatic sites**				
1	173 (69.5)	37 (55.2)	61 (70.1)	p=0.72
2	61 (24.5)	24 (35.8)	21 (24.1)	
≥ 3	15 (6.0)	6 (8.9)	5 (5.8)	
**Metastatic sites**				
Bone	180 (72.3)	46 (68.7)	53 (60.9)	p=0.05
Viscera	75 (30.1)	29 (43.3)	30 (34.5)	p=0.25
Soft tissue	84 (33.7)	25 (37.3)	31 (35.6)	p=0.67
**Previous therapies – metastatic setting**				
ET	76 (30.5)	26 (38.8)	41 (47.1)	p=0.02
CHT	23 (9.2)	5 (7.5)	6 (6.9)	
ET - CHT	150 (60.2)	36 (53.7)	40 (46.0)	
**Number of prior ET – metastatic setting**				
1	170 (75.2)	47 (75.8)	57 (70.4)	P=0.48
2	51 (22.6)	14 (22.6)	22 (27.2)	
≥ 3	5 (2.2)	1 (1.6)	2 (2.5)	
**Number of prior CHT – metastatic setting**				
1	90 (52.0)	21 (51.2)	28 (60.9)	P=0.06
2	43 (24.9)	10 (24.4)	16 (34.8)	
≥ 3	40 (23.1)	10 (24.4)	2 (4.6)	

^*^ Cochran-Mantel-Haenszel Statistics (Based on Table Scores) was used.

ET=endocrine therapy; CHT=chemotherapy.

### Treatment exposure and clinical activity

Median duration of EVE treatment was 28,5 weeks (95% CI 19,0 – 33,8) in patients ≥ 65 years and 24,4 weeks (95% CI 19,2 – 33,2) in those aged ≥ 70 years, lower, but not statistically significant (p=0.41), in both cases than what observed in the younger population (31,5 weeks, 27,0 – 35,3).

Calculation of Dose-Intensity (DI) has been already described in the main paper [10].

The distribution of EVE DI in the population used for the present analysis was calculated on 147 out of the 154 identified patients and was as follows: DI ≤ 5 mg/day, N= 26 (17,6%); DI 5.1 – 7,5 mg/day, N=48 (32,7%); DI > 7,5 mg/day, N=73 (49,6%).

Median EVE DI was > 7,5 mg/day in the majority of the patients (49,6%), even when fewer patients aged ≥ 65 years received the highest DI in comparison to younger patients (49,6% vs. 66,8%).

In patients aged ≥ 65 years, median duration of EVE treatment according to DI was 24,8 (95% CI 16,0 – 33,2), 23,5 (95% CI 16,6 – 38,5) and 27,7 (95% CI 19,0 – 35,9) weeks, without any difference among the 3 groups (χ^2^=0.23, p=0.62).

In patients aged 70 years or more, the distribution of patients according to the different levels of DI was 20,7%, 29,9% and 49,4% respectively. More patients in this group started EVE at the dose of 5 mg (6, 6,9%) in comparison to the other two groups.

In patients aged ≥ 70 years, median duration of EVE treatment according to DI was 24,9 (95% CI 24,0 – 33,2), 20.1 (95% CI 13,8 – 54,1) and 27,6 (95% CI 15,6 – 38,8) weeks, with no difference among the 3 groups (χ^2^=0.73, P=0.39).

**Figure 2:**
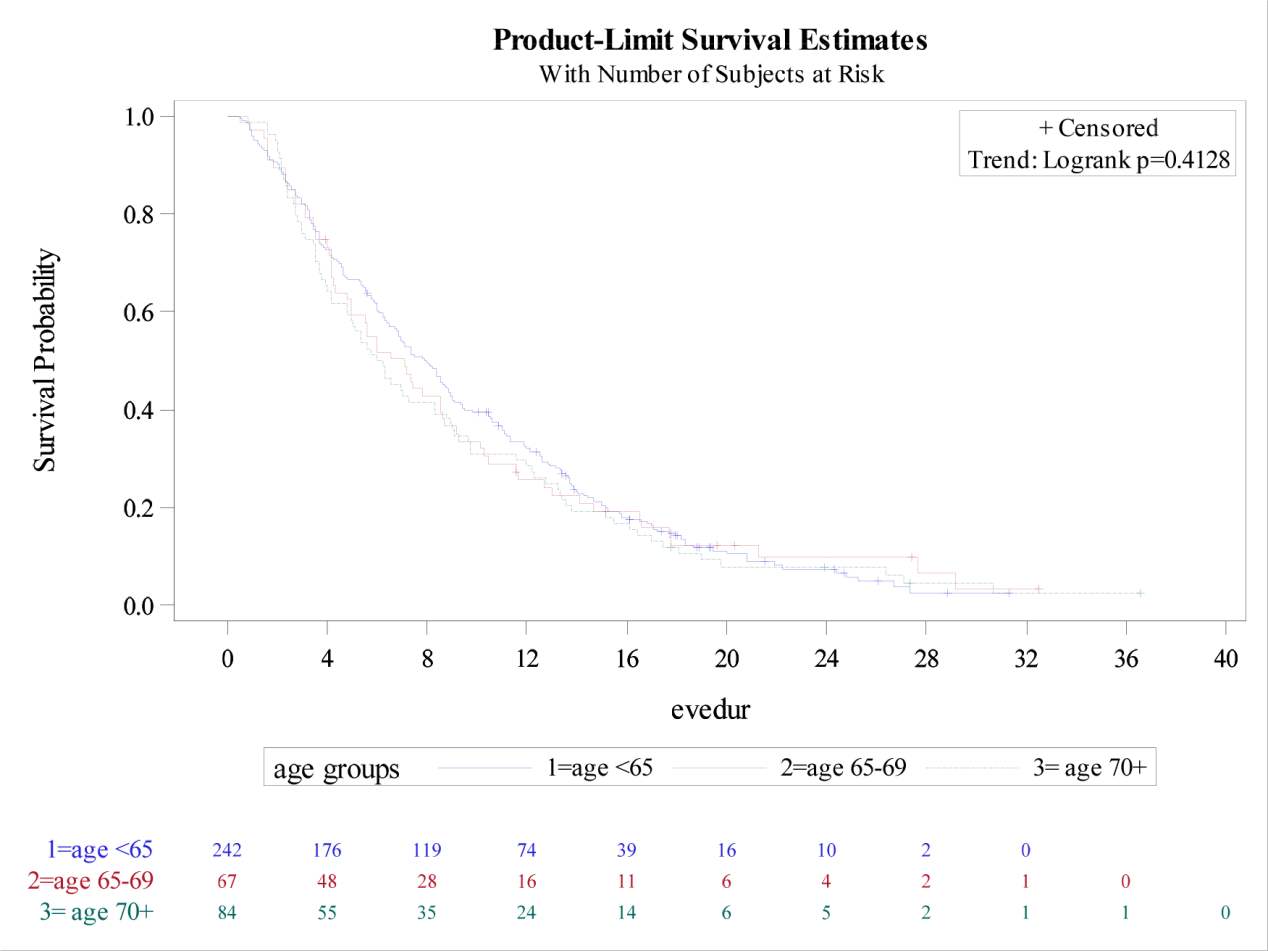
Time on everolimus treatment by age group

Data regarding median duration of EVE treatment by age groups are shown in Figure 2.

In the multivariate analysis, none of the variables analysed (ER status, DI, previous Fulvestrant, previous chemotherapy, site of metastases) showed statistical evidence of association with EVE treatment duration, with the exception of the number of metastatic sites, which was found to be associated with a lower duration of EVE treatment for both elderly populations (age ≥ 65 years: HR=0.77, 95% CI 0.59-1.00; age ≥ 70 years: HR=0.57, 95% CI 0.39-0.84).

ORR was observed in 48 patients (31,2%): according to DI, 5 pts (19,2%), 15 pts (31,3%), and 28 pts (38,4%) obtained an objective response. No significant differences have been observed among the 3 groups in terms of ORR (P=0.74), or DCR (P=0.45). Details according to age groups are summarized in Table 2.

**Table 2:** Efficacy data according to age groups

	Age < 65 years	Age 65 - 69 years	Age ≥ 70 years	p value^*^
Median Time on EVE treatment (weeks)	31.5 (27.0 – 35.3)	28.5 (19.0 – 33.8)	24.4 (19.2 – 33.2)	

Response	N (%)	N(%)	N(%)	
**CR**	11 (4.3)	5 (7.4)	3 (3.5)	
**PR**	64 (25.6)	15 (22.4)	25 (29.4)	
**SD**	65 (26.0)	19 (28.4)	26 (30.6)	
**ORR**	75 (30.0)	20 (29.8)	28 (32.9)	0.74
**DCR**	143 (57.2)	39 (58.2)	54 (63.5)	0.45

^*^ p value was calculated by Cochran-Mantel-Haenszel Statistics (Based on Table Scores) CR=Complete Response; PR=Partial response; SD=Stable Disease; ORR=Overall Response Rate; DCR=Disease Control Rate.

### Safety

All patients have been included in the safety analysis. One-hundred thirty-eight (89,7%) patients experienced an adverse event (AE) of any grade. Treatment-related Grade 3–4 AEs occurred in 55 patients (35,7%). Main Grade 3-4 toxicities were: stomatitis (10,9%), rash (5,8%) and non-infectious pneumonitis (NIP) (3,6%). Five EVE-related deaths, defined as death while on treatment, or within 30 days from the end of therapy, occurred: 4 in the group aged ≥ 70 years, the last patient was aged 67 years. Three patients have received EVE for a very long period of time (198, 201 and 240 days) and have showed PR in the first two cases and SD in the latter one. All these patients started the treatment at the dose of 10 mg/day: the first patient aged 67 years have received the full dose for 18 days and subsequently stopped the treatment for 53 days due to toxicity, she had no comorbidities and never had a response. The second patient was aged 70 years, received 201 days of full dose and died while on treatment for different causes. The third patient aged 72 years received 240 days of EVE full dose, 180 days at the reduced dose (5 mg/day) and had 80 days of drug suspension: she died 21 days after EVE discontinuation. The fourth patient was aged 72 years, presented multiple comorbidities and received 92 days of full dose EVE, dying 2 days after treatment discontinuation. The last patient was aged 80 years, received 198 days of full dose EVE, had a treatment suspension for 13 days and died 18 days after EVE permanent discontinuation due to PD.

Specifically, in patients ≥ 70 years, main Grade 3-4 toxicities were stomatitis (11,1%, median duration 15 days, range 3 - 120), fatigue (4,9%), anaemia (4,9%), NIP (3,7%) and rash (3,7%), without any difference according to DI.

Details of toxicity of the patients aged ≥ 65 years and of those aged ≥ 70 years are summarized in Table 3, together with the observed toxicity in patients aged < 65 years.

**Table 3:** Toxicity according to age groups

Adverse events	Aged < 65 years (N=250)		Aged 65 - 69 years (N=67)		Aged ≥ 70 years (N=87)	
	Any Grade n(%)	Grade 3-4 n(%)	Any Grade n(%)	Grade 3-4 n(%)	Any Grade n(%)	Grade 3-4 n(%)
Stomatitis	118 (47.2)	23 (9.2)	42 (62.7)	6 (8.9)	46 (56.8)	9 (11.1)
Fatigue	72 (28.8)	7 (2.8)	26 (38.8)	-	36 (44.4)	4 (4.9)
Rash	51 (20.4)	1 (0.4)	15 (22.4)	5 (7.4)	20 (24.7)	3 (3.7)
Hyper-cholesterolemia	41 (16.4)	-	11 (16.4)	-	11 (13.6)	-
Hyper-glycaemia	39(15.6)	1 (0.4)	6 (8.9)	1 (1.5)	15 (18.5)	1 (1.2)
Peripheral oedema	39 (15.6)	5 (2.0)	4 (5.9)	-	15 (18.5)	3 (3.7)
Anaemia	34 (13.6)	7 (2.8)	8 (11.9)	2 (2.9)	15 (18.5)	4 (4.9)
NIP	33 (13.2)	8 (3.2)	16 (23.9)	2 (2.9)	14 (17.3)	3 (3.7)
Liver toxicity	31 (12.4)	5 (2.0)	7 (10.4)	1 (1.5)	9 (11.1)	1 (1.2)
Diarrhoea	27 (10.8)	2 (0.8)	11 (16.4)	3 (2.2)	9 (11.1)	-
Weight loss	24 (9.6)	1 (0.4)	5 (7.4)	-	10 (12.3)	2 (2.5)
Neutropenia	24 (9.6)	8 (3.2)	3 (4.5)	-	4 (4.9)	1 (1.2)
Thrombocytopenia	23 (9.2)	2 (0.8)	3 (4.5)	-	9 (11.1)	2 (2.5)
Infections	22 (8.8)	4 (1.6)	11 (16.4)	1 (0.7)	5 (6.2)	-
Hyper-triglyceridemia	21 (8.4)	-	8 (11.9)	-	7 (8.6)	-
Nausea	19 (7.6)	-	7 (10.4)	-	7 (8.6)	-
Taste alteration	10 (4.0)	2 (0.8)	9 (13.4)	-	9 (11.1)	1 (1.2)
Vomiting	6 (2.4)	-	1 (1.5)	-	2 (2.5)	-
Electrolyte variations	5 (2.0)	2 (0.8)	2 (2.9)	-	2 (2.5)	-

## DISCUSSION

Median duration of EVE treatment was 28.5 weeks (95% CI 19.0 – 33.8) in patients ≥ 65 years and 24,4 weeks (95% CI 19,2 – 33,2) in those aged ≥ 70 years, lower in both cases than what observed in the younger population (< 65 years: 31.5 weeks, 27.0 – 35.3). Fewer patients aged ≥ 65 years received the highest EVE Dose-Intensity in comparison to younger patients (49,6% vs. 66,8%). Grade 3–4 toxicities occurred in 55 patients (35,7%), mainly stomatitis (10,9%), rash (5,8%) and non-infectious pneumonitis (NIP) (3,6%). Some toxicities, such as weight loss and anaemia were peculiarly observed in patients aged ≥ 70 years.

In the elderly subset of the BALLET expanded-access trial [10] (N=563, 26.3%), 95.2% of patients experienced at least one AE. The most common any grade AEs in elderly versus non-elderly patients were stomatitis (55.5% versus 51.9%), asthenia (28.5% versus 20.7%) and decreased appetite (22.4% versus 13.7%); the most frequent grade 3 or 4 AEs were stomatitis (12.3% versus 8.3%), asthenia (5.7% versus 2.9%) and hyperglycemia (4.6% versus 2.3%). NIP was reported in 11.2% of elderly versus 8.9% of non-elderly patients.

In the BOLERO-2 trial [4], patients older than 65 years of age were 275 (38,0%), while patients ≥ 70 were 164 (22,7%): the addition of EVE to EXE improved PFS regardless of age (hazard ratio, 0.59 [≥ 65 years] and 0.45 [≥70 years]). Adverse events of special interest (all grades) that occurred more frequently with EVE than with placebo included stomatitis, infections, rash, pneumonitis, and hyperglycemia. Elderly EVE-treated patients had similar incidences of these AEs as did younger patients but had more on-treatment deaths. Mouth-washes, as suggested by Rugo et Al, is strongly recommended [11]

Altogether these data provide a unique opportunity to determine the efficacy and safety of EVE in combination with EXE in these underserved elderly subsets. In addition to these previous trials, the EVA study provides the same opportunity with data coming from the real-life evidence [9].

To our knowledge, the EVA study is the largest real-life study which has reported efficacy and safety results, explored the potential correlation between EVE Dose Intensity and ORR or DCR and described the efficacy results in special subgroups of patients, namely those previously treated with Fulvestrant or chemotherapy.

To investigate the role of EVE in elderly patients and mainly to verify if it can be a safe treatment in this special population, we focused in this analysis on patients aged 65 years or more.

A pre-specified analysis of the BOLERO-2 trial done on 275 patients aged ≥ 65 years [8] concluded that the advantage in terms of PFS by adding EVE to EXE is independent of age and that adverse events (AEs) of special interest such as stomatitis, infections, rash, pneumonitis, and hyper-glycaemia had similar incidence as in younger patients, even if elderly ones had more on-treatment deaths. The Authors concluded that careful monitoring and appropriate dose reductions or interruptions for AE management are recommended during treatment with EVE in this patient population.

We observed a median duration of EVE treatment in patients aged ≥ 65 years of 28.5 weeks, lower than the one observed in the whole population of the EVA study (31,0 weeks) but similar to the one described in the sub-group analysis of BOLERO-2 (26,9 weeks); same results have been obtained for patients aged ≥ 70 years, when compared to the population described in the exploratory analysis of BOLERO-2 (24,4 vs. 23,2 weeks).

The difference observed between younger and older patients in the EVA study could be related to different reasons:

1) higher percentage of elderly patients treated with the lowest DI in comparison to younger patients (DI ≤ 5 mg/day: 17,6% vs. 13,2%) and lower rate of patients who have received the highest DI (> 7,5 mg/day: 49,6% vs. 66,8%). As previously reported [10], we found a potential correlation between low DI and lower duration of treatment, even if not statistically significant. However, the possibility of confounding because of poor performance status, physical condition or treatment discontinuation due to AEs cannot be excluded.
2) Higher rate of patients aged 70 years or more who started EVE at the dose of 5 mg.

In terms of Grade 3-4 key-toxicities, no important differences have been observed in elderly patients when compared to younger ones. However, some other toxicities, such as anaemia (4,9%), rash (3,7%), thrombocytopenia (2,5%) and weight loss (2,5%) seems to be peculiar of these groups of patients. Similarly, taste alteration (any grade) was reported in a higher percentage of elderly patients in comparison to younger ones (13,3% vs 4,0%).

Decreased weight has been reported as a potential AE for EVE [4] [12]. Weight loss after EVE-EXE treatment may be attributed in part to concomitant stomatitis, decreased appetite, and nausea, which are also commonly reported AEs for EVE, even if observed that the incidence of this adverse event was more frequently observed in the elderly populations. The careful and proactive management of stomatitis and anorexia may help avoiding significant weight loss during EVE therapy, as already recommended by different Authors [8] [13].

The pattern of toxicity observed in EVA elderly population is very close to what described in the sub-group analyses of the BOLERO-2 study. This finding is of great importance, because, as with most clinical trials in advanced breast cancer, BOLERO-2 required participants to have a good performance status and to have an adequate bone marrow, renal, and hepatic function—criteria that may have excluded very elderly patients with multiple comorbidities and/or myelosuppression from previous chemotherapy.

Grade 3-4 AE incidence is similar in the elderly and the overall populations of different trials, such as RECORD-1 in metastatic renal cell carcinoma patients and BOLERO-2 and EVA studies in breast cancer, supporting the overall tolerability of EVE regardless of age [4] [8] [12].

In our opinion, optimizing the benefit of EVE-EXE combination in elderly patients should not necessary include strict patients’ selection: our results from real-life suggest that there isn’t a higher incidence of toxicities in comparison to what observed in the registration study population, even if the availability of data regarding the optimal management of peculiar toxicities were available at the moment of EVA study beginning, but not during BOLERO-2 trial.

On the contrary, proactive management of AE risk in the context of comorbidities common to the elderly population remains a key issue for patients’ selection, as well as a careful monitoring of AEs during treatment is strongly recommended, in order to facilitate early diagnosis and appropriate management [9].

Recently, Freedman et Al [1] brilliantly reviewed the efficacy and safety in older patient subsets in studies of endocrine monotherapy versus combination therapy in patients with HR+/HER2- advanced breast cancer. They reported that in the second-line setting, older patients had median PFS of 6,8 and 9,9 months with Everolimus + Exemestane and palbociclib + Fulvestrant, respectively, and younger patients had median PFS of 8,1 and 9,5 months, respectively. Tolerability was worse for combination therapy versus monotherapy. No age-related differences in discontinuations were observed for CDK4/6 inhibitors, although a higher rate of treatment discontinuations were observed for patients ≥ 70 years receiving Everolimus + Exemestane. AE rates were similar in age-stratified subsets.

What does this sub-group analysis from the EVA study add to today’s treatment scenario for elderly HR+ patients with advanced breast cancer?

1) Understanding how to optimally manage cancer in older patients has become increasingly important as the population ages and the prevalence of breast cancer among older patients is increasing; other Authors [14] underlined that, although it is reassuring that the benefits of combination therapy were observed regardless of age in most of the recent trials concerning the role of targeted agents in combination with endocrine therapy, they acknowledge that these older patient subsets may not be fully representative of older patients with breast cancer in the general population, as patients with poor performance status or significant comorbidities were excluded from enrolment: this analysis from the EVA study provides further and in some cases more reliable information from the clinical practice and could be of help in reassuring clinicians regarding the use of EVE in safe conditions.
2) Data regarding the duration of EVE treatment, even if lower than the one described in younger patients, together with the similar rate of peculiar toxicities confirm that EVE-EXE should be a useful option of treatment also for elderly or very elderly patients: in our opinion, this option cannot be excluded *a priori* only in relation to patient’s age and remains a valid 2^nd^-line option, thus delaying the start of chemotherapy, often not easily manageable in older patients, mainly due to treatment compliance, toxicity or discontinuation due to AE [4].
3) Analysis of toxicity pattern suggest that in the everyday clinical practice clinicians have to monitor some peculiar aspects, such as food intake to prevent weight loss and to discuss with the patients and their care-givers which symptoms or signs must be monitored at home, in addition to the classical ones, such stomatitis.
4) Careful monitoring and implementation of appropriate EVE dose modifications for AEs must be considered for elderly patients, even if it seems that there is no reason to start EVE treatment at the lower dose.
5) In the changing scenario of treatments for HR+ ABC patients, EVE-EXE remains at the moment a potential alternative to chemotherapy after endocrine treatment failure.

## MATERIALS AND METHODS

### Patients

Patients aged ≥ 65 years were selected from the database of the EVA study, which is a multicentre retrospective cohort study, collecting data of 404 HR+ ABC patients who received EVE-EXE combination between July 2013 and December 2015 in 38 Oncology Centres in Italy. Data were collected via electronic database. Baseline information included patient’s age at metastatic diagnosis, comorbidities, breast cancer history, (date of stage at initial diagnosis, any adjuvant and/or neoadjuvant therapy), hormone and HER2 status, number and sites of metastases. Physicians were requested to provide a fully comprehensive description of previous endocrine treatments and chemotherapy including the number of previous treatments, as described in the main paper. The study was approved by the Ethical Committees of all Centres; all patients signed a written informed consent before clinical data were collected. Patients eligible for the present analysis were female, ≥ 65 years, with documented HR+ locally advanced or metastatic breast cancer, previously treated or not with other drugs for the metastatic disease, among which EVE-EXE was chosen by the physician, according to the clinical situation of the patient. Data retrieval included disease characteristics, hormone receptor and HER2 status, sites of metastases and tumour biology, as well as previous therapies received both in the adjuvant and the metastatic setting. Patients who were included into the BALLET trial or into other interventional EVE studies were excluded. For the purpose of the present pre-planned analysis, with particular respect to concomitant diseases at the moment of EVE-EXE start, comorbidities were collected grouped by apparatus (for example: under the term “cardiovascular” all kinds of disease attributable to cardiac and vascular districts have been included). In consideration of the data collection method and that the study didn’t aim to find correlations between comorbidity and efficacy or safety results, we decided to only provide a description of type and number of pre-existing medical conditions. Baseline patients’ and tumour characteristics, efficacy and safety results of patients aged ≥ 65 years, subdivided by cut off aged 70 or older, have been analysed and compared to those of younger ones.

### Treatment plan

No treatment plan was provided *a priori*, due to the observational nature of the study. Physicians were asked to identify all consecutive patients who fitted the pre-specified criteria of the study and to collect patients’ data from the clinical records in an electronic case-report-form (CRF) dedicated to the study. As described in the main paper [9], the effective Dose-Intensity (DI) of EVE therapy per patient was calculated adding the number of days at 10 mg plus those at 5 mg plus the number of days without any dose (0 mg), the sum was then divided per the total administered dose for the effective number of treatment days, including the interruption periods. We subsequently identified three groups of patients according to the following DIs: A) ≤ 5mg/day; B) 5.1 – 7.5 mg/day and C) > 7.5 mg/day.

### Clinical outcomes

All measures of clinical outcomes were based on physician's evaluation and no central review was planned. The primary end-point of this analysis was the duration of EVE treatment in weeks. Secondary end points were: overall response rate (ORR) and disease control rate (DCR) by RECIST 1.1, toxicity (according to CTC criteria Version 2.0) and EVE Dose-Intensity.

Patients who had not progressed, were censored at the data cut-off date (January, 2017).

### Statistical considerations

Demographic data, baseline characteristics of patients and disease, and treatment information were summarised with standard summary statistics (median, and range for continuous data, relative and absolute frequencies for categorical data). Relationship of these variables with age and response were tested by mean of a Cochran-Mantel-Haenszel chi square test for trend. Time to event analysis was described by Kaplan Meier approach and association with baseline patient and tumour characteristics was analysed by stratified log-rank test and proportional hazard model. Sample size was reported and detailed in the main paper; however, it was sufficient to obtain a quite precise description of chosen statistics and a good fit with the Cox model. 200 events overall were deemed sufficient for modelling up to 10 variables, including age at EVE start up [15].

## Abbreviations

(EVE): Everolimus
(EXE): Exemestane
(ET): endocrine therapy
(HER2−): human epidermal growth factor receptor 2-negative
(ABC): advanced breast cancer
(CDK 4/6-Is): cyclin-dependent kinase 4/6 inhibitors
(PFS): progression-free survival
(ORR): Overall Response Rate
(DI): Dose-Intensity
(AE): adverse event

## Appendix 1

List of Co-Authors

Cicchiello F.^2^, Riva F.^2^, Ballerio A.^3^, Coccato S.^6^, Valerio M.R.^**7**^, De Martini P.^8^, Cocciolone V.^10^, Gianni L.^11^, Michelotti A.^12^, Campidoglio S.^14^, Dester M.^15^, Pizzuti L.^19^, Caggia F.^20^, Ferrari L.^21^, Girelli S.^22^, Bedolis R.^24^, Alù M.^25^, De Laurentiis M.^26^, Bordin E.^28^, Porpiglia M.^29^ Santini D.^30^, Bozzoli E.^31^, Marchese A.^32^.

^2^ Oncology Unit, ASST Monza, Monza, Italy.

^3^ Oncology Unit, ASST della Valle Olona - Presidio Ospedaliero di Saronno, Saronno, Italy.

^6^ Oncology Unit, AULSS 3 Mirano, Italy.

^7^ Oncology Department, Policlinico di Palermo Paolo Giaccone Palermo, Italy.

^8^ Oncology Unit 2 - Città della Salute e della Scienza di Torino, Torino, Italy.

^10^ Dipartimento di Scienze Cliniche Applicate e Biotecnologiche (DISCAB) - Università Degli Studi Dell'Aquila.

^11^ Oncology Unit Rimini Azienda USL Romagna, Rimini, Italy.

^12^ Oncology Unit 2, Azienda Ospedaliera Universitaria Pisa via Roma 67 Pisa, Italy.

^14^ Oncology Unit, Ospedale Sacro Cuore di Gesù, Fatebenefratelli, Benevento, Italy.

^15^ Breast Unit, ASST di Cremona, Italy.

^19^ Oncology Unit 2, Istituto Nazionale Tumori Regina Elena – IFO, Roma, Italy.

^20^ Oncology Unit, Policlinico University Hospital of Modena, Italy.

^21^ Oncology Unit, ASL di Frosinone Osp. "SS. Trinità", Italy.

^22^ Oncology Unit, ASST Fatebenefratelli Sacco Presidio Ospedaliero Fatebenefratelli, Milano, Italy.

^24^ Oncology Departement, Ospedale di Gallarate ASST Valle Olona, Gallarate, Italy.

^25^ Oncology Unit, ARNAS Civico Palermo, Palermo, Italy.

^26^ National Cancer Institute "Fondazione Giovanni Pascale", Napoli, Italy.

^28^ Ospedale Martini della ASL "Città di Torino.

^29^ Dipartimento Universitario Ginecologia e Ostetricia 1, Ospedale S. Anna Torino, Italy.

^30^ Oncology Unit, Università Campus Bio-Medico, Roma, Italy.

^31^ Oncology Unit 1, Istituto Regina Elena – IFO, Roma, Italy.

^32^ Oncology Unit, Ospedale Sacro Cuore di Gesù, Fatebenefratelli, Benevento, Italy.

## References

[1] Freedman RA, Foster JC, Seisler DK, Lafky JM, Muss HB, Cohen HJ, Mandelblatt J, Winer EP, Hudis CA, Partridge AH, Carey LA, Cirrincione C, Moreno-Aspitia A (2017). Accrual of Older Patients With Breast Cancer to Alliance Systemic Therapy Trials Over Time: protocol A151527. J Clin Oncol.

[2] Basche M, Barón AE, Eckhardt SG, Balducci L, Persky M, Levin A, Jackson N, Zeng C, Vranas P, Steiner JF (2008). Barriers to enrollment of elderly adults in early-phase cancer clinical trials. J Oncol Pract.

[3] Biganzoli L, Wildiers H, Oakman C, Marotti L, Loibl S, Kunkler I, Reed M, Ciatto S, Voogd AC, Brain E, Cutuli B, Terret C, Gosney M (2012). Management of elderly patients with breast cancer: updated recommendations of the International Society of Geriatric Oncology (SIOG) and European Society of Breast Cancer Specialists (EUSOMA). Lancet Oncol.

[4] Baselga J, Campone M, Piccart M, Burris HA, Rugo HS, Sahmoud T, Noguchi S, Gnant M, Pritchard KI, Lebrun F, Beck JT, Ito Y, Yardley D (2012). Everolimus in postmenopausal hormone-receptor-positive advanced breast cancer. N Engl J Med.

[5] Finn RS, Martin M, Rugo HS, Jones S, Im SA, Gelmon K, Harbeck N, Lipatov ON, Walshe JM, Moulder S, Gauthier E, Lu DR, Randolph S (2016). Palbociclib and Letrozole in Advanced Breast Cancer. N Engl J Med.

[6] Hortobagyi GN, Stemmer SM, Burris HA, Yap YS, Sonke GS, Paluch-Shimon S, Campone M, Blackwell KL, André F, Winer EP, Janni W, Verma S, Conte P (2016). Ribociclib as First-Line Therapy for HR-Positive, Advanced Breast Cancer. N Engl J Med.

[7] Davidson A, Chia S, Olson R, Nichol A, Speers C, Coldman AJ, Bajdik C, Woods R, Tyldesley S (2013). Stage, treatment and outcomes for patients with breast cancer in British Columbia in 2002: a population-based cohort study. CMAJ Open.

[8] Pritchard KI, Burris HA, Ito Y, Rugo HS, Dakhil S, Hortobagyi GN, Campone M, Csöszi T, Baselga J, Puttawibul P, Piccart M, Heng D, Noguchi S (2013). Safety and efficacy of everolimus with exemestane vs. exemestane alone in elderly patients with HER2-negative, hormone receptor-positive breast cancer in BOLERO-2. Clin Breast Cancer.

[9] Cazzaniga ME, Airoldi M, Arcangeli V, Artale S, Atzori F, Ballerio A, Bianchi GV, Blasi L, Campidoglio S, Ciccarese M, Cursano MC, Piezzo M, Fabi A, on behalf of, and EVA Study Group (2017). Efficacy and safety of Everolimus and Exemestane in hormone-receptor positive (HR+) human-epidermal-growth-factor negative (HER2-) advanced breast cancer patients: new insights beyond clinical trials. The EVA study. Breast.

[10] Jerusalem G, Mariani G, Ciruelos EM, Martin M, Tjan-Heijnen VC, Neven P, Gavila JG, Michelotti A, Montemurro F, Generali D, Simoncini E, Lang I, Mardiak J (2016). Safety of everolimus plus exemestane in patients with hormone-receptor-positive, HER2-negative locally advanced or metastatic breast cancer progressing on prior non-steroidal aromatase inhibitors: primary results of a phase IIIb, open-label, single-arm, expanded-access multicenter trial (BALLET). Ann Oncol.

[11] Rugo HS, Seneviratne L, Beck JT, Glaspy JA, Peguero JA, Pluard TJ, Dhillon N, Hwang LC, Nangia C, Mayer IA, Meiller TF, Chambers MS, Sweetman RW (2017). Prevention of everolimus-related stomatitis in women with hormone receptor-positive, HER2-negative metastatic breast cancer using dexamethasone mouthwash (SWISH): a single-arm, phase 2 trial. Lancet Oncol.

[12] Porta C, Calvo E, Climent MA, Vaishampayan U, Osanto S, Ravaud A, Bracarda S, Hutson TE, Escudier B, Grünwald V, Kim D, Panneerselvam A, Anak O, Motzer RJ (2012). Efficacy and safety of everolimus in elderly patients with metastatic renal cell carcinoma: an exploratory analysis of the outcomes of elderly patients in the RECORD-1 Trial. Eur Urol.

[13] Rugo HS, Pritchard KI, Gnant M, Noguchi S, Piccart M, Hortobagyi G, Baselga J, Perez A, Geberth M, Csoszi T, Chouinard E, Srimuninnimit V, Puttawibul P (2014). Incidence and time course of everolimus-related adverse events in postmenopausal women with hormone receptor-positive advanced breast cancer: insights from BOLERO-2. Ann Oncol.

[14] Freedman RA, Tolaney SM (2018). Efficacy and safety in older patient subsets in studies of endocrine monotherapy versus combination therapy in patients with HR+/HER2- advanced breast cancer: a review. Breast Cancer Res Treat.

[15] Peduzzi P, Concato J, Feinstein AR, Holford TR (1995). Importance of events per independent variable in proportional hazards regression analysis. II. Accuracy and precision of regression estimates. J Clin Epidemiol.

