# Correction 2: Everolimus (EVE) and exemestane (EXE) in patients with advanced breast cancer aged ≥ 65 years: new lessons for clinical practice from the EVA study

**DOI:** 10.18632/oncotarget.26431

**Published:** 2018-11-30

**Authors:** Marina Cazzaniga, Claudio Verusio, Mariangela Ciccarese, Alberto Fumagalli, Donata Sartori, Maria Rosaria Valerio, Mario Airoldi, Gabriella Moretti, Corrado Ficorella, Valentina Arcangeli, Lucrezia Diodati, Alberto Zambelli, Antonio Febbraro, Daniele Generali, Mirco Pistelli, Ornella Garrone, Antonino Musolino, Patrizia Vici, Michela Maur, Lucia Mentuccia, Nicla La Verde, Giulia Bianchi, Salvatore Artale, Livio Blasi, Michela Piezzo, Francesco Atzori, Anna Turletti, Chiara Benedetto, Maria Concetta Cursano, Alessandra Fabi, Vittorio Gebbia, Alessio Schirone, Raffaella Palumbo, Antonella Ferzi, Antonio Frassoldati, Claudio Scavelli, Luca Clivio, Valter Torri

**Affiliations:** ^1^ Research Unit Phase I trials, ASST Monza, Monza, Italy; ^2^ Oncology Unit, ASST Monza, Monza, Italy; ^3^ Oncology Unit, ASST della Valle Olona-Presidio Ospedaliero di Saronno, Saronno, Italy; ^4^ Oncology Unit, Ospedale “Vito Fazzi” di Lecce, Lecce, Italy; ^5^ Oncology Unit, Ospedale Moriggia Pelascini, Gravedona, Italy; ^6^ Oncology Unit, AULSS 3, Mirano, Italy; ^7^ Oncology Department, Policlinico di Palermo Paolo Giaccone, Palermo, Italy; ^8^ Oncology Unit 2-Città della Salute e della Scienza di Torino, Torino, Italy; ^9^ Oncology Unit, IRCCS Arcispedale S. Maria Nuova, Reggio Emilia, Italy; ^10^ Dipartimento di Scienze Cliniche Applicate e Biotecnologiche (DISCAB)-Università Degli Studi Dell'Aquila, L'Aquila, Italy; ^11^ Oncology Unit Rimini Azienda USL Romagna, Rimini, Italy; ^12^ Oncology Unit 2, Azienda Osp edaliera Universitaria Pisa via Roma 67, Pisa, Italy; ^13^ Oncology Unit, ASST Papa Giovanni XXIII, Bergamo, Italy; ^14^ Oncology Unit, Ospedale Sacro Cuore di Gesù, Fatebenefratelli, Benevento, Italy; ^15^ Brest Unit, ASST di Cremona, Cremona, Italy; ^16^ Oncology Unit, AOU Ospedali Riuniti Umberto I-GM Lancisi-G Salesi, Ancona, Italy; ^17^ Oncology Unit, AOS Croce e Carle Ospedale di Insegnamento, Cuneo, Italy; ^18^ Oncology Unit, Azienda Ospedaliero-Universitaria di Parma, Parma, Italy; ^19^ Oncology Unit 2, Istituto Nazionale Tumori Regina Elena–IFO, Roma, Italy; ^20^ Oncology Unit, Policlinico University Hospital of Modena, Modena, Italy; ^21^ Oncology Unit, ASL di Frosinone Osp. “SS. Trinità”, Sora, Italy; ^22^ Oncology Unit, ASST Fatebenefratelli Sacco Presidio Ospedaliero Fatebenefratelli, Milano, Italy; ^23^ Oncology Unit 1, Fondazione IRCCS Istituto Nazionale Tumori, Milano, Italy; ^24^ Oncology Departement, Ospedale di Gallarate ASST Valle Olona, Gallarate, Italy; ^25^ Oncology Unit, ARNAS Civico Palermo, Palermo, Italy; ^26^ National Cancer Institute “Fondazione Giovanni Pascale”, Napoli, Italy; ^27^ Struttura Complessa di Oncologia Medica Azienda Ospedaliero-Universitaria Cagliari, Cagliari, Italy; ^28^ Oncology Unit, Ospedale Martini della ASL Città di Torino, Torino, Italy; ^29^ Dipartimento Universitario Ginecologia e Ostetricia 1, Ospedale S. Anna Torino, Turin, Italy; ^30^ Oncology Unit, Università Campus Bio-Medico, Roma, Italy; ^31^ Oncology Unit 1, Istituto Regina Elena–IFO, Roma, Italy; ^32^ Oncology Unit, Ospedale La Maddalena, Palermo, Italy; ^33^ Oncolgy Department, Istituto Scientifico Romagnolo per lo Studio e la Cura dei Tumori (IRST) IRCCS, Meldola, Italy; ^34^ Oncology Unit, IRCCS ICS Maugeri, Pavia, Italy; ^35^ Oncology Unit, ASST OVEST Milanese, Presidio di Legnano, Legnano, Italy; ^36^ Oncology Unit, Az Ospedaliero Universitaria di Ferrara, Ferrara, Italy; ^37^ Oncology Unit, Ospedale “S. Cuore di Gesù”, Gallipoli, Italy; ^38^ IRCCS-Istituto di Ricerche Farmacologiche “Mario Negri”, Milano, Italy

**This article has been corrected:** The correct author name is given below:

**Michela Piezzo**

Original article: Oncotarget. 2018; 9:31877-31887. https://doi.org/10.18632/oncotarget.25874

